# Supplementary material for: Joint morphogenetic cells in the adult mammalian synovium
Source: Nat Commun. 2017 May 16;8:15040. doi: 10.1038/ncomms15040 (PMC5493527; doi:10.1038/ncomms15040)
Supplement: Supplementary Information — Supplementary Figures, Supplementary Tables and Supplementary References [file ncomms15040-s1.pdf]

SUPPLEMENTARY INFORMATION

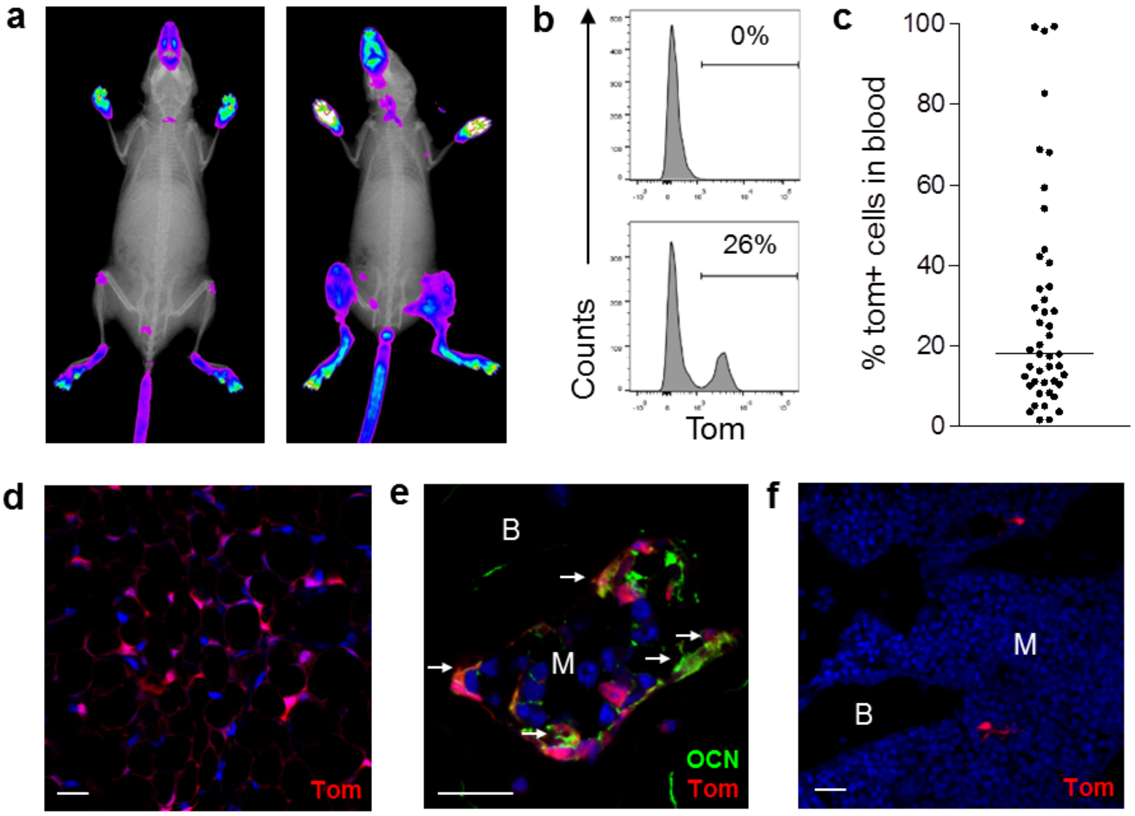

Supplementary Figure 1. *Gdf5*-lineage tracing. (**a**) *In vivo* imaging to detect Tom fluorescence in non-leaky (left) and leaky (right) adult *Gdf5-Cre*;*Tom* mice (n=3 leaky and 6 non-leaky mice). (**b**) Flow cytometry analysis of peripheral blood from non-leaky (top) and leaky (bottom) *Gdf5-Cre*;*Tom* mice. Analysis of 116 mice revealed 45 leaky mice (39%), in keeping with the previously reported 37% of leaky mice in the *Gdf5-Cre*;*LacZ* model[1]. (**c**) Percentage of Tom^+ cells in peripheral blood of 45 leaky mice. Line indicates median. (**d**) Tom^+ (red) cells in infrapatellar fat pad (n=4). Nuclei were counterstained with DAPI (blue). (**e**) Tom^+ (red) osteoblasts (arrows) in epiphyseal bone stained for osteocalcin (OCN; green) (n=2). Nuclei were counterstained with DAPI (blue). (**f**) Tom^+ (red) cells were rarely observed in metaphysis (n=3). Nuclei were counterstained with DAPI (blue). B, bone; M, marrow. Scale bars in all panels: 20 µm.

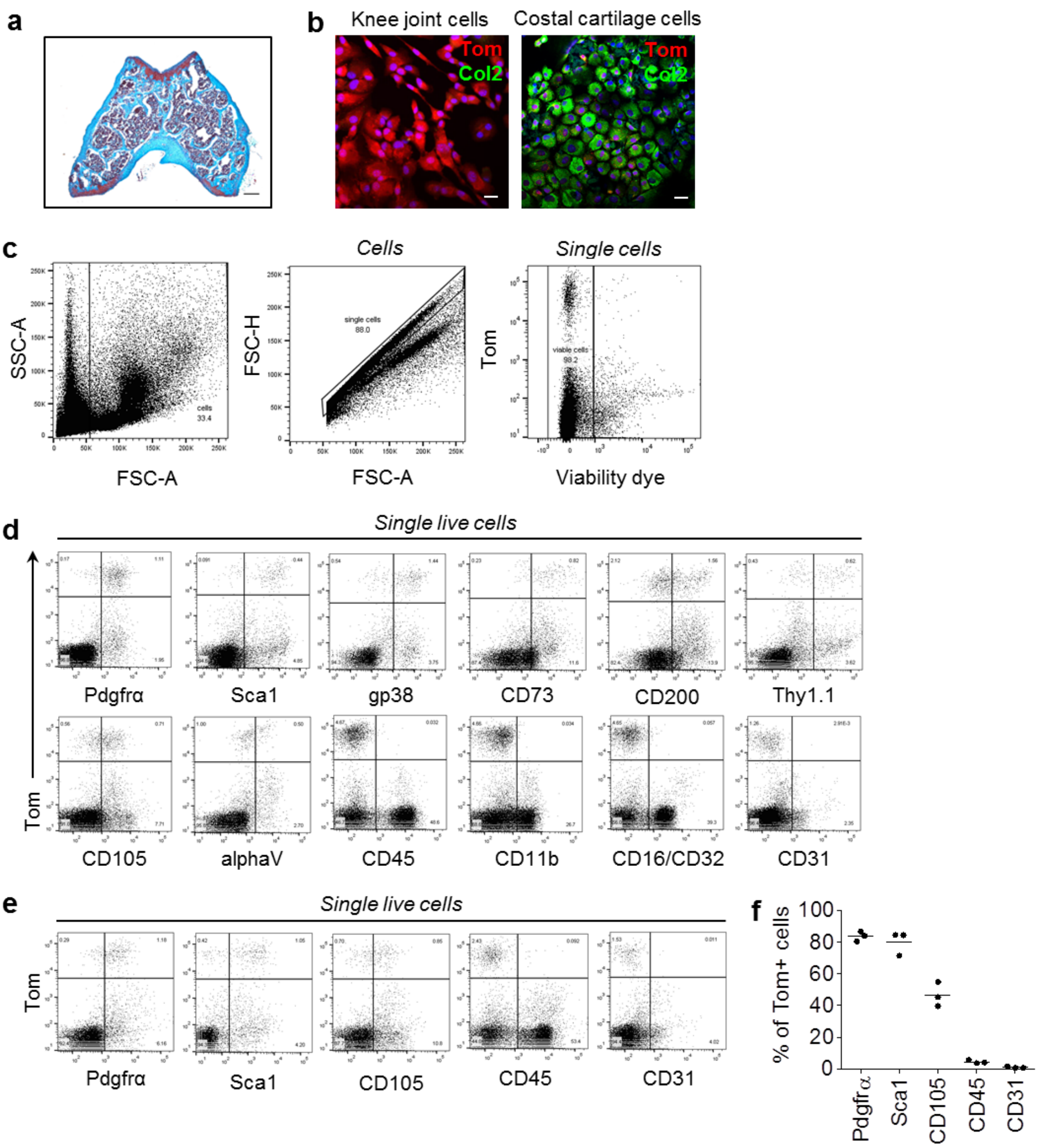

Supplementary Figure 2. Phenotypic analysis of *Gdf5*-lineage cells using flow cytometry. (**a**) Safranin O staining of a knee joint after 50 min collagenase digestion to isolate synovial cells. Cross-section through the femoral epiphysis shows articular cartilage and subchondral bone marrow remaining in the digested joint (n=9). (**b**) Immunostaining for Col2 (green) and Tom (red) of *Gdf5-Cre*;*Tom* cells at p0 isolated from knee joint using 50 min collagenase digestion (left), and costal cartilage using overnight collagenase digestion as positive control (right). Col2-expressing chondrocytes were detectable in the costal chondrocyte culture but not the synovial cell culture (n=2; 2 experiments). Nuclei were counterstained with DAPI (blue). Scale bars: 20 µm. (**c**) Gating strategy to identify single live cells within fresh cell isolates. Erythrocytes and debris were gated out based on Forward-Side Scatter profile; doublets and aggregates were gated out based on Forward Scatter parameters; dead cells were excluded based on viability dye staining. (**d,e,f**) Cell surface marker analysis of freshly isolated cells from (**d**) 3-5-month-old (n=3-18; pooled data from 7 experiments), and (**e,f**) 10-12-month-old (n=3) *Gdf5-Cre*;*Tom* mice. (**d,e**) Representative flow cytometry plots showing Tom and marker positivity. (**f**) Percentage of Tom$^+$ cells from 10-12-month-old mice (n=3) expressing the cell surface markers tested.

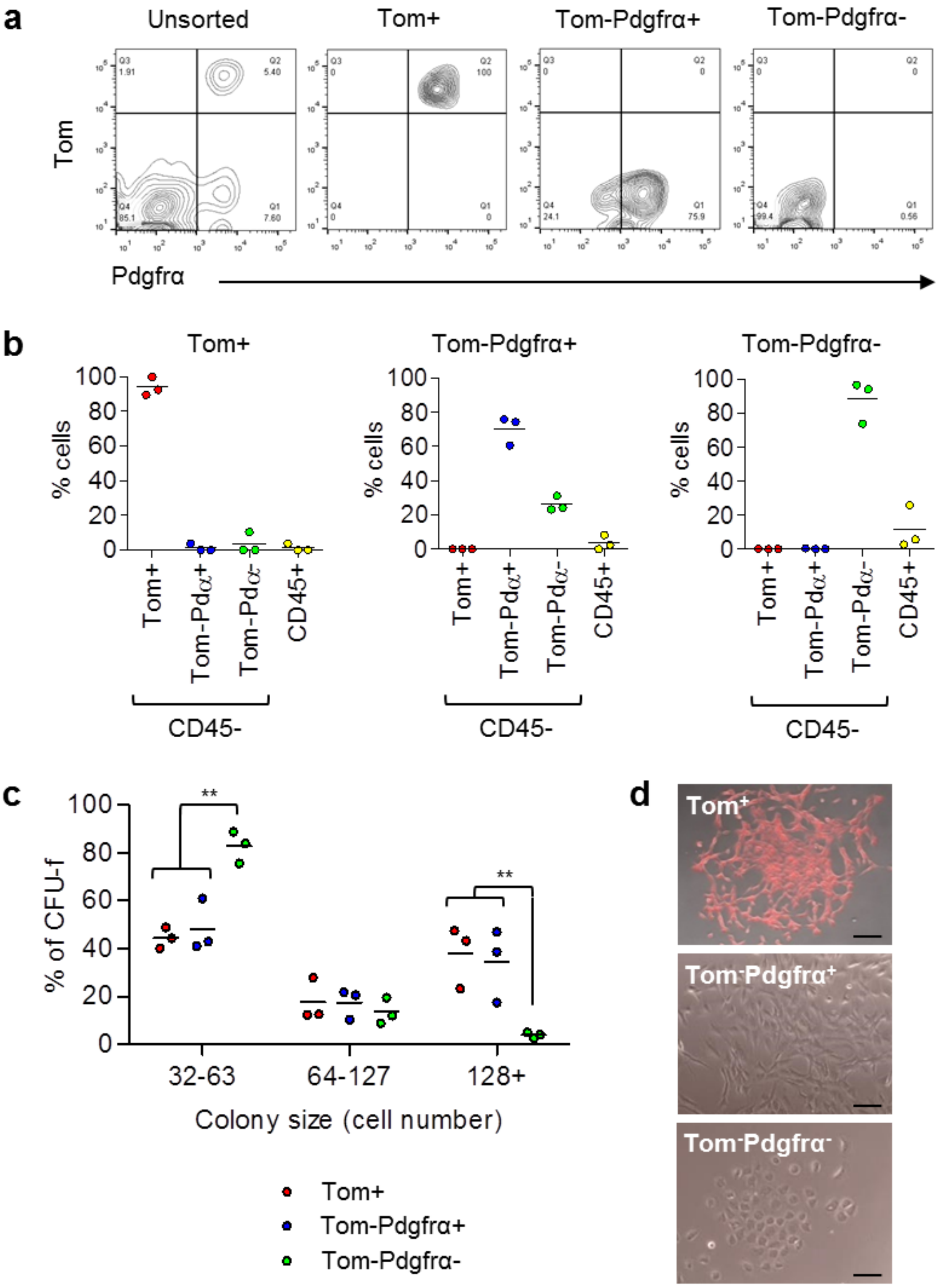

Supplementary Figure 3. Colony-forming-unit activity of *Gdf5*-lineage cells. (**a**) Representative flow plots of the different sorted cell fractions analysed immediately after sorting to assess purity (n=3). (**b**) Degree of enrichment achieved for the Tom^{+}, Tom^{-}Pdgfrα^{+}, and Tom^{-}Pdgfrα^{-} fractions. (**c**) Colony size distribution, showing that Tom^{+} and Tom^{-}Pdgfrα^{+} stromal cells gave rise to significantly larger colonies on average compared to double-negative stromal cells. **p<0.01; n=3; two-way ANOVA with Bonferroni post-test. (**d**) Representative images of colonies at time of analysis. Red: Tom fluorescence.

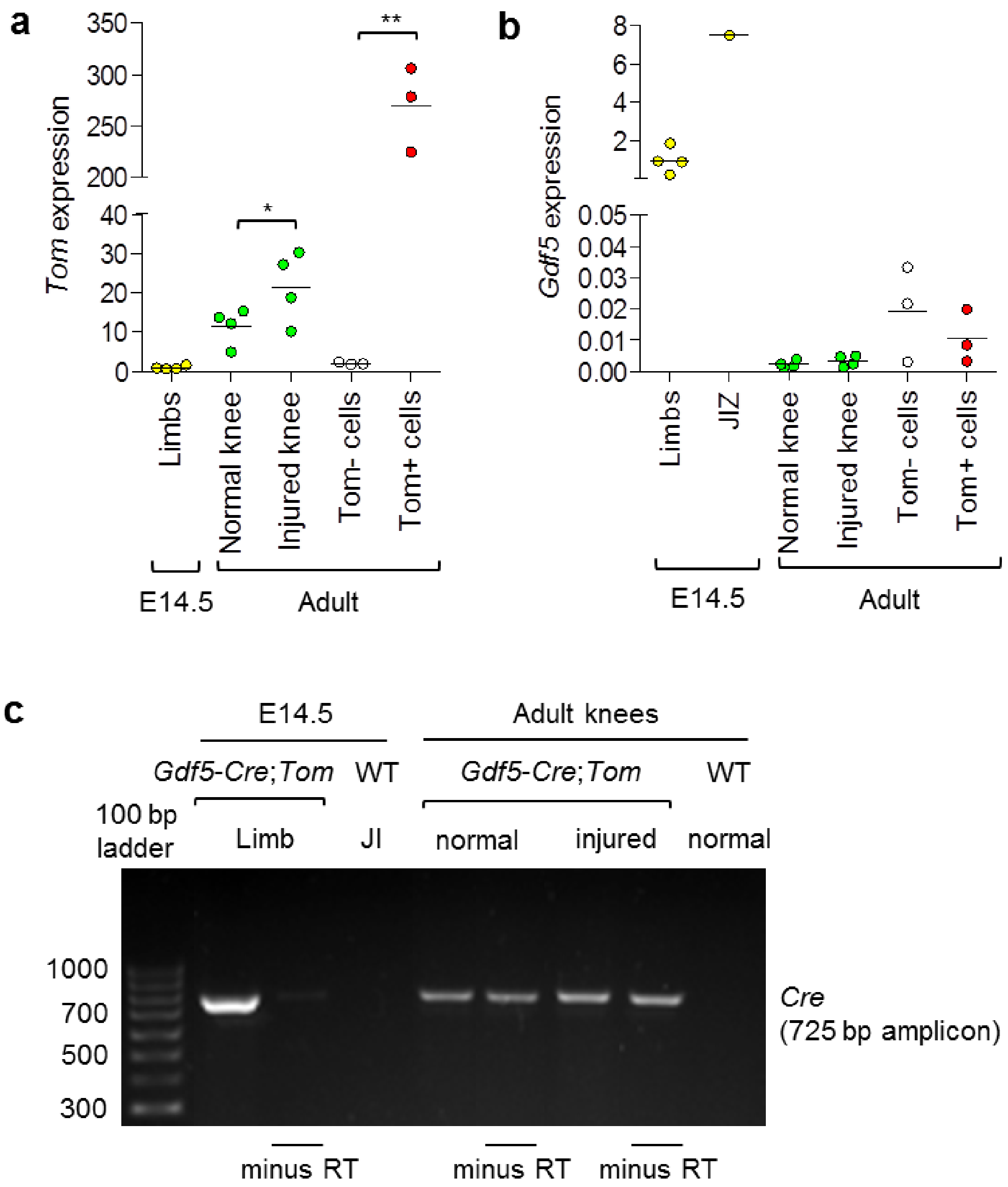

Supplementary Figure 4. Validation of *Gdf5-Cre*;*Tom* model for lineage tracing from joint interzone. Expression of (**a**) *Tom* and (**b**) *Gdf5* determined by quantitative RT-PCR, and (**c**) *Cre* detected by RT-PCR, in *Gdf5-Cre*;*Tom* E14.5 embryonic whole limbs (n=4), joint interzone (JI) of WT E14.5 embryo (n=1), soft tissues of normal and injured adult *Gdf5-Cre*;*Tom* mouse knees at 2-4 days after cartilage injury (n=4), and Tom$^+$ and Tom$^-$ sorted cells cultured for 44 d prior to RNA extraction (n=3). Data in (**a,b**) was normalised to *Gapdh* and expressed relative to E14.5 limbs. *p=0.0303; **p=0.0077 (Student's t-test). Data in (**c**) is shown as compilation image of lanes on same gel. Numbers on left indicate DNA ladder band sizes (in basepairs). Minus RT indicates reverse transcriptase was omitted from the sample, revealing that the amplified product detected in adult knees originated from genomic *Cre*. Taken together, data show that while *Tom* expression in adult knee soft tissues was >10 fold higher than E14.5 embryonic limbs, *Gdf5* expression was negligible and comparable to Tom$^-$ sorted cells which had remained negative for Tom during culture. In addition, no upregulation of *Gdf5* or *Cre* expression was detected after injury despite a ~2-fold increase in *Tom* expression.

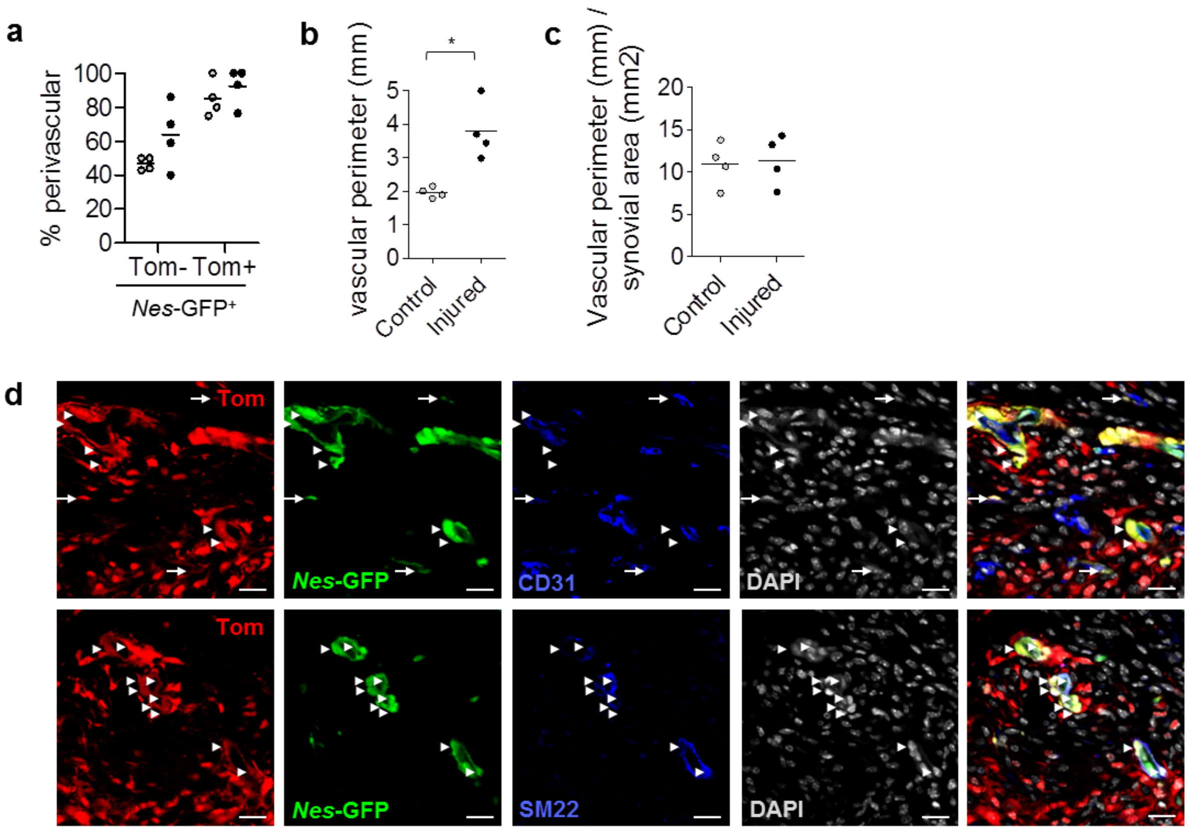

Supplementary Figure 5. Relationship between *Gdf5*-lineage and *Nes*-GFP$^+$ cells in adult synovium. (**a**) Percentage of labeled cells that were perivascular showing Tom$^+$*Nes*-GFP$^+$ cells to be almost exclusively perivascular in both control and injured knee synovium (n=4). (**b,c**) The total vascular perimeter (in mm) in injured synovium was greater compared to control (**b**; *p=0.0252; n=4; Student's t-test with Welch's correction) but not different when normalised for synovial area (**c**; n=4). (**d**) Separate channel images for Fig. 3i. Adjacent histological sections stained with CD31 (top; blue) or SM22 (bottom; blue) showing Tom$^+$ (red) and *Nes*-GFP$^+$ (green) double-labeled cells (appearing yellow) in injured knee synovium localising predominantly around arterioles and expressing the smooth muscle marker SM22 (arrowheads) with some Tom$^+$*Nes*-GFP$^+$ cells found around other CD31$^+$ vessels (arrows) (n=3). Nuclei were counterstained with DAPI (blue). Scale bars: 20 µm.

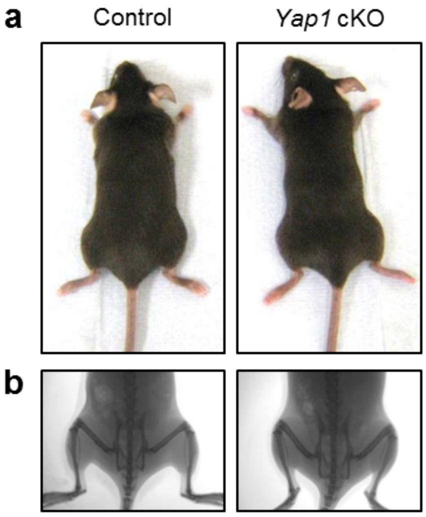

Supplementary Figure 6. Conditional *Yap1* KO in *Gdf5* lineage. Adult *Gdf5-Cre*;*Yap1*$^{fl/fl}$;*Tom* (*Yap1* cKO) mice displayed no obvious phenotypic abnormalities compared to littermate controls. (**a**) Brightfield images. (**b**) X-ray images of hindlegs.

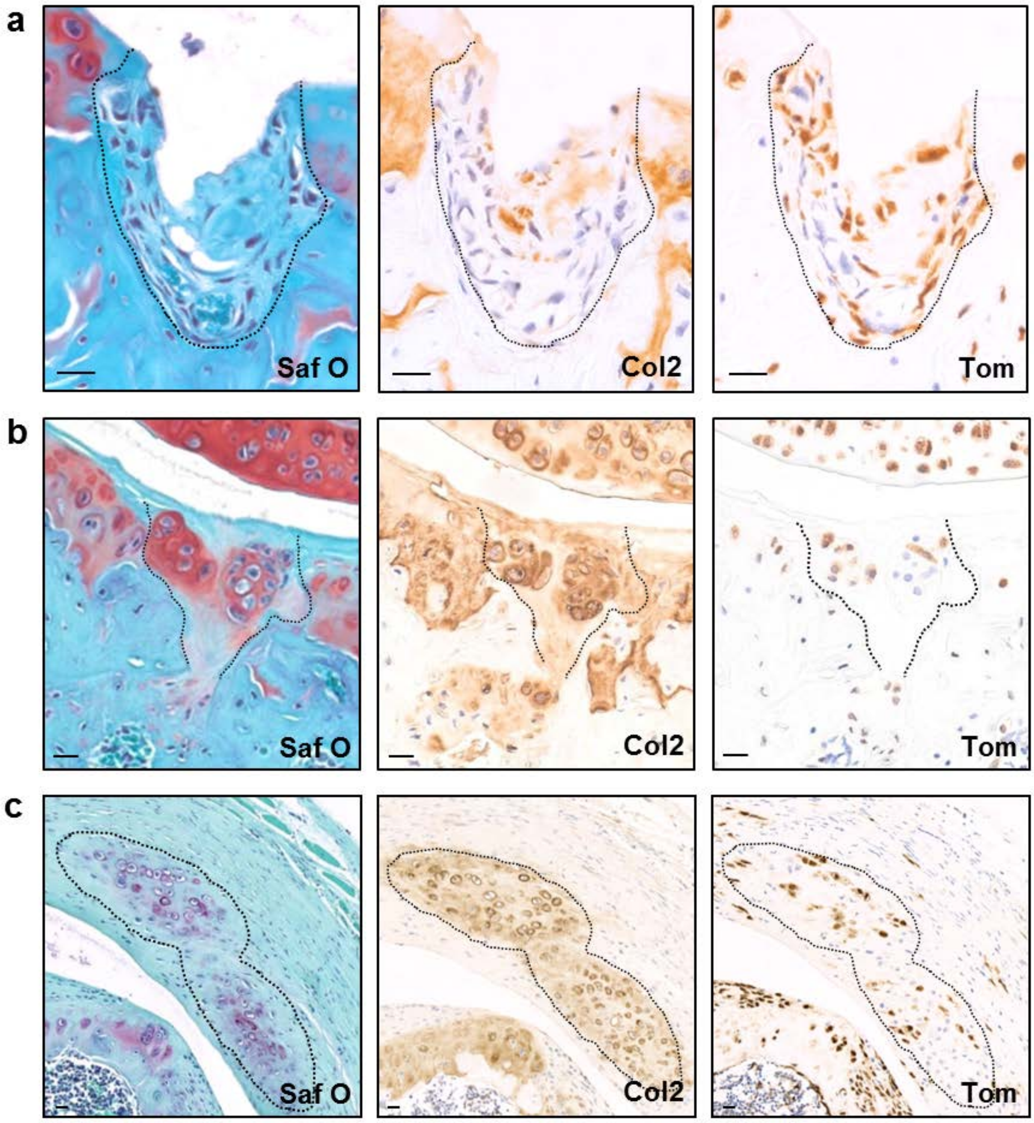

Supplementary Figure 7. Contribution of *Gdf5*-lineage cells to cartilage formation after injury. Tom+ cells in (**a**) fibrous-like (n=8) and (**b**) cartilage-like repair of the articular cartilage (n=4), and (**c**) ectopic cartilage-like tissue in synovium (n=4). Consecutive histological sections (from left to right) were stained with Safranin O/fast green (Saf O), collagen type II (Col2; brown, with haematoxylin counterstain in blue), or Tom (brown, with haematoxylin counterstain in blue). Note the presence of reporter-negative chondrocytes in (**b,c**). Scale bars: 20 µm.

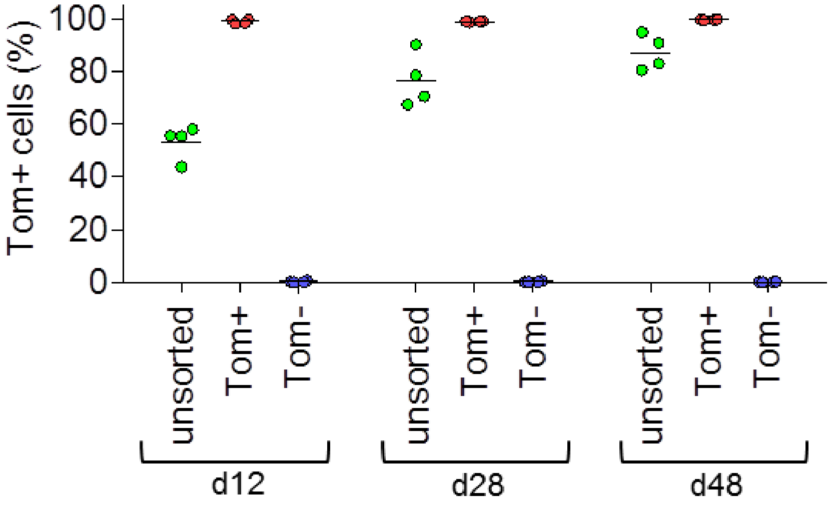

Supplementary Figure 8. Culture expansion of *Gdf5*-lineage cells. Tom$^+$ cells outgrew Tom$^-$ cells during routine culture expansion of unsorted knee joint cells from *Gdf5-Cre*;*Tom* mice (n=4; representative of 2 experiments).

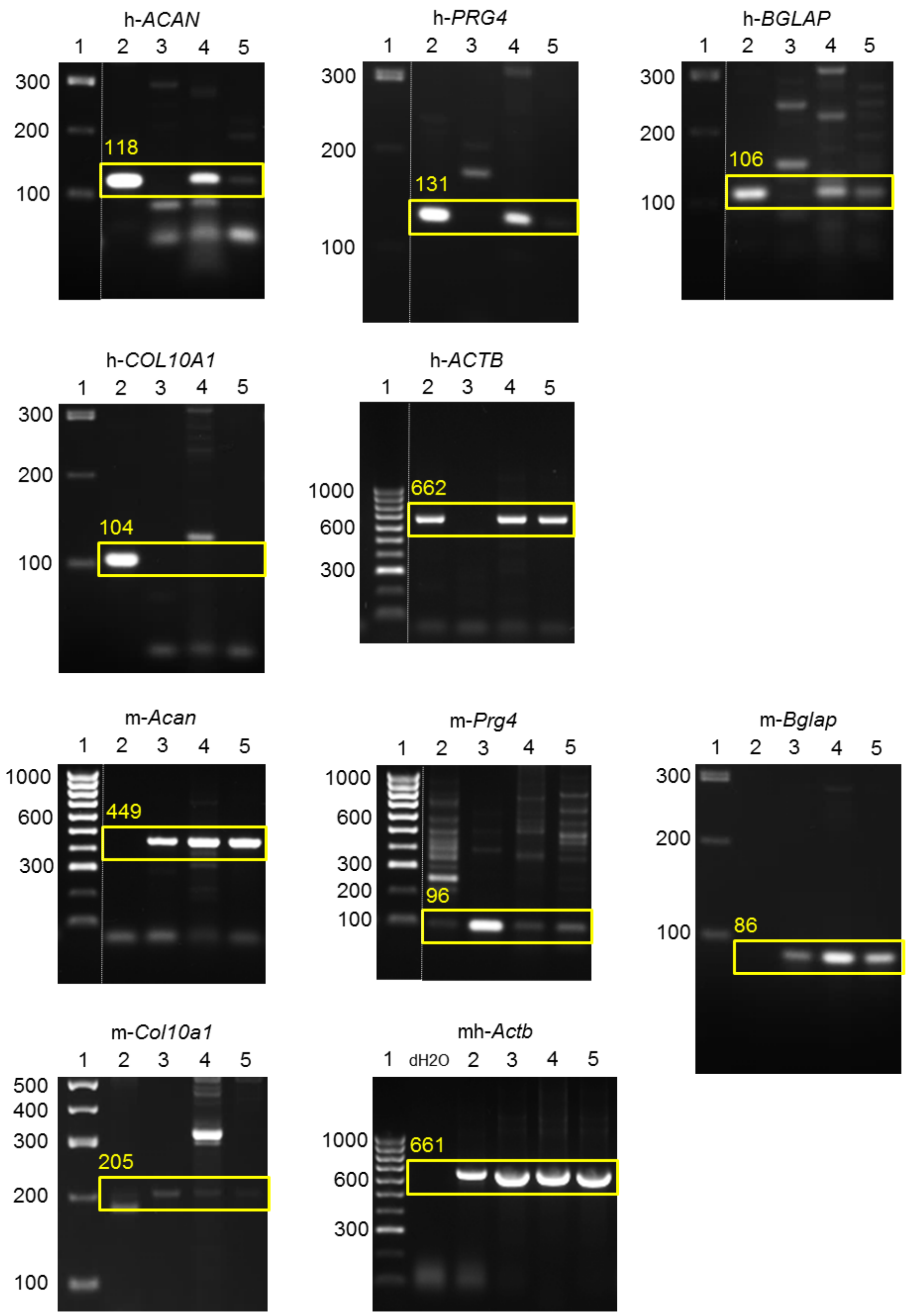

Supplementary Figure 9. Gels of RT-PCR analysis shown in Fig. 8f. Lanes show: 1) 100 bp DNA ladder; 2) human positive control; 3) mouse knee joint positive control; 4) human synovial MSC implant; 5) human dermal fibroblast implant. h: primer pair specific for human; m: primer pair specific for mouse; mh: primer pair detects both mouse and human. The human positive control (lane 2) served as negative control for detection of mouse genes; the mouse positive control (lane 3) served as negative control for detection of human genes; dH2O was used as negative control for detection of mh-*ACTB*. Dotted line indicates that lanes on the gel between the DNA ladder and the relevant samples were omitted from the image. Numbers on left indicate DNA ladder band sizes (in basepairs). Correct amplicon sizes are indicated in yellow on the gels (in basepairs).

Supplementary Table 1. Antibodies for immunostaining.

Antibody	Clone	Manufacturer	Catalogue Number	Conjugation
RFP	polyclonal	Rockland	600-401-379	unconjugated
mCherry	polyclonal	Sicgen antibodies	AB0081-200	unconjugated
IdU	IIB5	Abcam	ab8955	unconjugated
CldU	BU1/75	Abcam	ab6326	unconjugated
Collagen type 2	polyclonal	Abcam	ab21291	unconjugated
Osteocalcin	polyclonal	AbD Serotec	70601815	unconjugated
Cadherin-11	polyclonal	Invitrogen Corp.	71-7600	unconjugated
CD140a/Pdgfrα	Polyclonal	Abcam	ab61219	unconjugated
Lubricin	polyclonal	Abcam	ab28484	unconjugated
CD31	polyclonal	Abcam	ab28364	unconjugated
Gremlin	polyclonal	R&D Systems	AF956	unconjugated
CD16/CD32	polyclonal	R&D Systems	AF1460	unconjugated
Yap	polyclonal	Novus Biologicals	NB110-58358	unconjugated
Ki67	MIB-1	Dako	M7240	unconjugated
Transgelin/SM22	polyclonal	R&D systems	AF7886	unconjugated
CD68	KP1	eBioscience	14-0688	unconjugated
CD55/DAF	polyclonal	R&D Systems	AF2009	unconjugated
Goat anti-rabbit IgG	polyclonal	Vector Labs	BA-1000	biotinylated
Donkey anti-rabbit IgG	polyclonal	ThermoFisher	A-21206	Alexa Fluor®488
Donkey anti-rabbit IgG	polyclonal	ThermoFisher	A-21207	Alexa Fluor®594
Goat anti-rabbit IgG	polyclonal	ThermoFisher	A-11012	Alexa Fluor®594
Donkey anti-rabbit IgG	polyclonal	Abcam	ab150067	Alexa Fluor®647
Donkey anti-goat IgG	polyclonal	ThermoFisher	A-11055	Alexa Fluor®488
Donkey anti-goat IgG	polyclonal	Abcam	ab150136	Alexa Fluor®594
Donkey anti-mouse IgG	polyclonal	Abcam	ab150109	Alexa Fluor®488
Goat anti-mouse IgG	polyclonal	ThermoFisher	A-11029	Alexa Fluor®488
Goat anti-rat IgG	polyclonal	ThermoFisher	A-21094	Alexa Fluor®633
Donkey anti-sheep IgG	polyclonal	ThermoFisher	A-21448	Alexa Fluor®647

Supplementary Table 2. Antibodies for flow cytometry.

Antibody	Clone	Manufacturer	Catalogue Number	Conjugated
CD45	30-F11	BD Biosciences	563890	BV421
CD31	390	eBioscience	48-0311	eFluor450
CD16/CD32	2.4G2	BD Biosciences	558636	APC
CD11b	M1/70	BD Biosciences	553312	APC
CD140a/Pdgfrα	APA5	BD Biosciences	562774	BV421
Sca1	D7	BD Biosciences	560654	APC-Cy7
CD105	MJ7/18	BD Biosciences	562761	AF647
gp38	8.1.1	Biolegend	127410	APC
AlphaV/CD51	RMV-7	eBioscience	MCA2461A647T	AF647
Thy1.1	OX-7	BD Biosciences	563770	BV421
CD200	OX90	eBioscience	50-5200	eFluor660
CD73	TY/23	BD Biosciences	561543	AF647
LepR	polyclonal	Bioss	bs-0109R-A647	AF647

Supplementary Table 3. Primers for RT-PCR.

Gene	Species	Primer	Sequence (5' to 3')	Amplicon (bp)
Tom	n/a	Fw	CTGTTCCTGTACGGCATGG	196
		Rev	GGCATTAAAGCAGCGTATCC	
Gdf5	Mouse	Fw	CGGACTGTAACCCCAAAAGGA	373
		Rev	TCCGTAAGATCCGCAGTTCAG	
Gapdh	Mouse	Fw	GTGAAGGTCGGTGTGAACG	250
		Rev	ATTTGATGTTAGTGGGGTCTCG	
Cre	n/a	Fw	GCCTGCATTACCGGTCGATGCAACGA	725
		Rev	GTGGCAGATGGCGCGGCAACACCATT	
ACAN	Human	Fw	CGGTCTACCTCTACCCTAACCA	118
		Rev	GGTGTGCCACCCTCTTCTT	
Acan	Mouse	Fw	CGTTGCAGACCAGGAGCAAT	449
		Rev	TCTTCTGCCCGAGGGTTCTA	
PRG4	Human	Fw	CTGGCCTGAATCTGTGTATTTTT	131
		Rev	GTGTCGTTTCTCCATACACTGG	
Prg4	Mouse	Fw	GCCACCTGCAACTGTGATTA	96
		Rev	GCGTCCTTTGCAGGAGAG	
BGLAP	Human	Fw	GGCGCTACCTGTATCAATGG	106
		Rev	TCAGCCAACTCGTCACAGTC	
Bglap	Mouse	Fw	AGACTCCGGCGCTACCTT	86
		Rev	CAAGCAGGGTTAAGCTCACA	
COL10A1	Human	Fw	CACCTTCTGCACTGCTCATC	104
		Rev	GGCAGCATATTCTCAGATGGA	
Col10a1	Mouse	Fw	GCATCTCCCAGCACCAGAAT	205
		Rev	TCTCCTCTTACTGGAATCCCTTT	
ACTB	Human	Fw	CCGACAGGATGCAGAAGGAG	662
		Rev	GGCACGAAGGCTCATCATTC	
Actb	Mouse + Human	Fw	TGACGGGGTCACCCACACTGTGCCCATCTA	661
		Rev	CTAGAAGCATTTGCGGTGGACGATGGAGGG	

Supplementary Reference

1. Rountree, R.B. *et al*. BMP receptor signaling is required for postnatal maintenance of articular cartilage. *PLoS. Biol.* **2**, e355 (2004).
